# *Legionella* Confer Survival Benefits to *Paramecium* Hosts by Inhibiting Phagocytosis

**DOI:** 10.1264/jsme2.ME25022

**Published:** 2025-10-31

**Authors:** Hiroko Kiyota, Kenta Watanabe, Hibiki Oyama, Masato Tachibana, Takashi Shimizu, Masahisa Watarai

**Affiliations:** 1 Laboratory of National BioResource Project Paramecium, Joint Faculty of Veterinary Medicine, Yamaguchi University; 2 Laboratory of Veterinary Public Health, Joint Faculty of Veterinary Medicine, Yamaguchi University; 3 Research Center for Thermotolerant Microbial Resources, Organization for Research Initiatives, Yamaguchi University

**Keywords:** *Legionella*, *Paramecium*, symbiotic relationship

## Abstract

*Legionella* survive in the natural environment by remaining within protist host cells. Many protist species, including *Paramecium* spp., are potential hosts for *Legionella*. However, the factors and mechanisms involved in the establishment of this relationship are unknown. The advantages gained by *Paramecium* spp. when they maintain *Legionella* are also unclear, and the existence of these relationships has not been confirmed. In the present study, feeding with *Legionella* increased the number of *Paramecium* cells over time. However, the growth-promoting effect of *Legionella* was weaker than that of *Klebsiella pneumoniae*, which is considered the optimal bacterial feed for *Paramecium*. Phagocytosis was strongly inhibited in *Paramecium* cells fed *Legionella*, indicating that this relationship prevents the uptake of harmful organisms. The inhibition of phagocytosis was also observed when *Paramecium* cells were treated with the *Legionella* culture supernatant. Despite the inhibition of phagocytosis, the presence of live* Legionella* within host cells allowed *Paramecium spp.* to survive and even increase in number, as observed earlier. This result suggests that *Legionella* support the survival of *Paramecium* hosts from a nutritional aspect. Although it is difficult to definitively state whether the relationship between *Legionella* and *Paramecium* hosts is completely mutualistic, the present results provide one rationale for defining their relationship.

Symbiosis between different organisms is a commonly observed relationship. The term “symbiosis” is most commonly defined in a narrow sense as a mutualistic relationship; however, there are many variations, such as commensalism, in which only one species benefits, and amensalism, in which one species is inhibited or harmed. The relationship between microorganisms and eukaryotes is a typical case, and many animals and plants have established various relationships with a wide variety of bacteria and fungi, including intestinal bacteria, resident skin microbiota, and mycorrhizal fungi. In recent years, research on symbiotic bacteria in the intestinal tract has been extensively conducted within the medical field. The findings obtained have demonstrated that intestinal symbiotic bacteria confer benefits not only in terms of nutrient metabolism, but also in the promotion of host immunity and in various aspects of host health (Hooper, 2009; Round and Mazmanian, 2009; Mazziotta *et al.*, 2023). In the case of non-mammalian species, symbiotic bacteria in insects, for example, have been shown to exert a significant impact on reproductive processes and directly affect phenotypic traits, such as color (Narita *et al.*, 2007; Tsuchida *et al.*, 2010; Landmann, 2019). Nevertheless, only a limited number of symbiotic bacteria are understood to possess these functions and significance. In many cases, including unidentified and unclassified species, the adaptive significance and biological benefits for both the host and symbiont have yet to be elucidated.

*Legionella*, the causative agent of legionellosis in humans, is a ubiquitous bacterial genus that is typically isolated from environmental soil and water, including engineered water systems (Fliermans *et al.*, 1981; Tachibana *et al.*, 2013; Cunha *et al.*, 2016; Orkis *et al.*, 2018). *Legionella* establish relationships with various protists, such as free-living amoebae and *Tetrahymena*, and survive and multiply in their intracellular environment (Rowbotham, 1980; Fields *et al.*, 1984). The major cause of the virulence of *Legionella* in humans is the ability to multiply intracellularly in macrophages, which is facilitated by the type IV secretion system (T4SS) and the many effectors that it secretes into host cells (Marra *et al.*, 1992; Berger and Isberg, 1993; Sadosky *et al.*, 1993). This activity also functions in a similar manner when bacteria survive within protists, their natural hosts, such as amoeba (Al-Quadan *et al.*, 2012; Richards *et al.*, 2013). These findings indicate that T4SS and effectors, the typical pathogenic factors of *Legionella* in humans, have their origins in a mechanism for establishing and maintaining relationships with protist hosts in the natural environment. Therefore, a study of the relationship between bacteria and their protist hosts may be critical for analyzing the pathogenicity of bacteria and obtaining insights into the control of legionellosis in humans.

*Paramecium* spp. are highly phagocytic and motile protists that live widely in natural and artificial freshwater environments. *Paramecium* may play a role in the environment as symbiotic hosts for various bacteria. For example, *Holospora* spp., Gram-negative *alpha-proteobacteria*, have been reported as nucleus-specific symbionts of *P. caudatum* (Gromov and Ossipov, 1981; Amann *et al.*, 1991; Fujishima *et al.*, 1991). They are highly specific to *P. caudatum* because *Holospora undulata* only invades the micronucleus and *H. obtusa* only invades the macronucleus, where they proliferate. We also found that another species of *Paramecium*, *Paramecium bursaria*, was a natural host for *Francisella novicida* (Watanabe *et al.*, 2022). However, the mechanisms of their symbiosis, their overall ecology, and their importance or impact as hosts in the natural environment remain unclear.

We previously reported that *Paramecium* spp., protists belonging to the ciliate class, were also potential natural hosts of *Legionella* in the environment (Watanabe *et al.*, 2016, 2018). We identified several *Legionella* genes involved in establishing a stable relationship with *Paramecium* hosts, as well as factors important for disrupting this relationship and causing cellular toxicity to the host cell (Watanabe *et al.*, 2016, 2018; Nishida *et al.*, 2018). Specifically, we revealed that TolC, the outer membrane protein of the type I secretion system, was essential for *L. pneumophila* to remain within *Paramecium* cells and to exhibit cytotoxicity (Nishida *et al.*, 2018). Furthermore, *lefA* was identified as a factor involved in cytotoxicity against *Paramecium* cells because it was highly expressed in some strains that kill *Paramecium* hosts, and this cytotoxicity was reduced in the deleted mutant strain (Watanabe *et al.*, 2016). It has yet to be established whether the relationship between *Paramecium* hosts and *Legionella* spp. may be described as a symbiotic relationship that benefits both sides and, thus, difficulties are associated with accurately describing their relationship. *Legionella* may obtain a transient living environment and growth niches by using *Paramecium* as a host. The high mobility and extensive distribution of *Paramecium* spp. may also facilitate the expansion of the range of existence of *Legionella* spp., which may be viewed as a benefit for the bacterium. On the other hand, the significance and benefits for *Paramecium* hosts of maintaining *Legionella* intracellularly have yet to be clarified.

Therefore, the present study exami­ned cellular changes that occur in *Paramecium* hosts that maintain *Legionella* intracellularly, as well as the potential benefits of this relationship to *Paramecium* spp. This ana­lysis was conducted with reference to previous studies on the symbiotic relationship between bacteria and their natural hosts.

## Materials and Methods

### Bacterial strains

*L. pneumophila* Philadelphia-1 (Phi-1), a human clinical isolate, Ofk308, an environmental water isolate (Tachibana *et al.*, 2013; Watanabe *et al.*, 2015), and their deletion mutant strains were maintained as frozen glycerol stocks and cultured at 37°C on N-(2-acetamido)-2-aminoethanesulfonic acid-buffered charcoal yeast extract agar or in the same medium without agar and charcoal (AYE) using glass test tubes. *Escherichia coli* strain DH5α and a non-pathogenic strain of *Klebsiella pneumoniae* were cultured in LB broth using glass test tubes or on LB containing 1.5% agar at 37°C. If necessary, chloramphenicol (10‍ ‍μg mL^–1^) or kanamycin (30‍ ‍μg mL^–1^) was included in the medium. Green fluorescent protein (GFP) or mCherry expression in *L. pneumophila* and *E. coli* was induced by adding isopropyl-β-d-thiogalactopyranoside (1‍ ‍mM) to AYE or LB.

### *Paramecium* host strains

*P. caudatum* RB-1 (PC042001A) was provided by the NBRP Paramecium Laboratory, Yamaguchi University with partial support by the National Bio-Resource Project of Ministry of Education, Culture, Sports, Science and Technology. *Paramecium* cells were cultured and maintained as previously described (Fujishima *et al.*, 1990). In brief, the culture medium used for *Paramecium *was 2.5% (w/v) fresh lettuce juice in Dryl’s solution (Dryl, 1959), which was inoculated with *K. pneumoniae* 1 day before use.

### Growth rate or cytotoxicity measurement

*Legionella* were liquid-cultured under the conditions described above for 48 h. The bacterial number was assessed by measuring optical density at 590‍ ‍nm and counting colony-forming units to calibrate the multiplicity of infection (MOI). Following the replacement of the medium with distilled water and the adjustment of bacterial counts, bacteria were added to *P. caudatum* RB-1 in 1.5-mL tubes at an MOI of 10^2^, 10^3^, 10^4^, or 10^5^. Similarly, *K. pneumoniae* cultured for 24‍ ‍h was added to RB-1. In experiments of mixed feeding, the following patterns were conducted: RB-1 was fed Phi-1, Ofk308, or a mixture of both strains in varying ratios from 1:1 to 10:1 at an Ofk308 MOI of 10^4^, and cultured at 25°C for 24 h. Another pattern is that RB-1 was fed *K. pneumoniae* or Phi-1 at an MOI of 10^4^. After 30‍ ‍min or 2 h, Ofk308 was fed to the host cells at the same MOI, which were cultured at 25°C for a further 24 h. Bacterial culture supernatants were prepared from *Legionella* and *E. coli* incubated at 37°C for 48‍ ‍h in AYE medium as described above. They were centrifuged at 5,000‍ ‍rpm for 15‍ ‍min, and the supernatants were then filtered through a 0.22-μm filter (Merck Millipore). These culture supernatants were added to RB-1 in a 1.5-mL tube at a final concentration of 0.1, 1, or 10%. The tubes were incubated at 25°C for 24–72 h. In experiments to confirm temperature tolerance, the same method was used to incubate the cells at 30°C and 37°C for 48 h. After the incubation, a 100-μL sample was collected from each group and the motility of *Paramecium* cells was confirmed. They were then fixed with 4% paraformaldehyde in PBS at room temperature for 10‍ ‍min. Samples were applied to glass slides with a 0.07-mm gap with a cover glass (Matsunami Glass) to count the number of cells. The number of viable cells with a normal morphology was quantified using microscopy. Cell counts are presented as relative values, with percentages based on the number of RB-1 cells at the beginning of the culture, which was defined as 100%.

### Fluorescence microscopy

GFP- or mCherry-expressing bacteria were washed with PBS and added to RB-1 at an MOI of 10^4^, followed by an incubation at 25°C for 2–24 h. In experiments of mixed feeding, RB-1 was fed a mixture of GFP-expressing Phi-1 and mCherry-expressing Ofk308 at a 1:1 ratio and an MOI of 10^4^ or mCherry-expressing Ofk308 2‍ ‍h after GFP-expressing Phi-1 feeding. To assess the phagocytic capacity of RB-1, 4.55×10^7^ particles of fluorescent microspheres (Fluoresbrite® YO Carboxylate Microspheres, 1.00‍ ‍μm; Polysciences) were added to RB-1, which was previously fed GFP-expressing *E. coli* or *Legionella* at an MOI of 10^4^ or treated with culture supernatants for 2 h, followed by an incubation at 25°C for 2 h. Samples were fixed with 4% paraformaldehyde in PBS at room temperature for 10‍ ‍min. The relative degree of fluorescence was calculated from the average of 10 *Paramecium* cells randomly selected from each sample. Fluorescent images were obtained, and the relative degree of fluorescence was evaluated using a FluoView FV100 confocal laser-scanning microscope (Olympus).

### Observations of the survival of *Paramecium* under the inhibition of phagocytosis

RB-1 was fed GFP-expressing Phi-1 at an MOI of 10^4^, the culture supernatant of Phi-1 at a final concentration of 0.1, 1, or 10% or AYE at a final concentration of 10%. Two and 48‍ ‍h later, to confirm the inhibition of phagocytosis, these cells were incubated with the same concentration of fluorescent beads as described above, followed by observations of cells 2‍ ‍h later. The relative degrees of fluorescence were calculated using the same method as described above. Survival rates were assessed at the designated time points in accordance with the method described above. Furthermore, at 24 or 48‍ ‍h post-treatment, *K. pneumoniae* was fed to the host cells at an MOI of 10^4^.

### Treatment of the culture supernatant

Each of the following treatments was performed on the Phi-1 culture supernatant collected using the above method. The separation of the culture supernatant into the <3‍ ‍kDa and >3‍ ‍kDa fractions was performed using an Amicon-Ultra-4 filter (Merck Millipore) according to the manufacturer’s instructions. The Proteinase K (Nacalai) treatment was conducted at a concentration of 1‍ ‍mg mL^–1^ at 37°C for 60 min, and the heat treatment was performed at 95°C for 30‍ ‍min. These culture supernatants were added to RB-1 at a final concentration of 10%. After 2 h, these cells were incubated at 25°C with the same concentration of fluorescent beads as described above, followed by observations of cells 2‍ ‍h later. The relative degrees of fluorescence are calculated using the same method as described above.

### Statistical ana­lysis

The Student’s *t*-test or multiple comparisons using the Tukey-Kramer test were used to evaluate the significance of differences. Significant differences between groups were accepted at *P*<0.05 or *P*<0.01. Data are presented as the average of triplicate samples from three identical experiments, and error bars represent standard deviations.

## Results

### Assessment of the growth rate of *Paramecium* cells

**Fig. 1.**
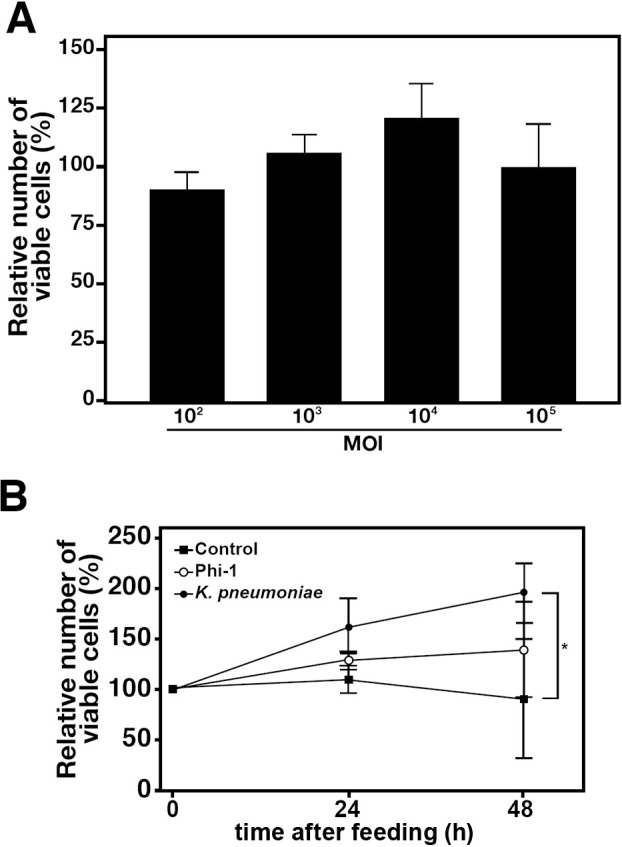
The growth rate of *Paramecium* hosts. The relative growth rate of *Paramecium* cells 48‍ ‍h after feeding with Phi-1 at varying MOIs (A) or 24 and 48‍ ‍h after feeding with *Klebsiella pneumoniae* and Phi-1 at an MOI of 10^4^ (B). Percentages are based on the number of *Paramecium* cells at the beginning of the culture, which was defined as 100%. Data are averages based on samples from three identical experiments, and error bars represent standard deviations. Significant differences from the control (without any feeding) are indicated by asterisks (*P<0.05).

To evaluate the growth rate of *Paramecium* host cells containing intracellular *Legionella*, cells were co-cultured with various numbers of Phi-1, and cell count variations were measured. Feeding was performed at an MOI of 10^2^–10^5^, and the number of *Paramecium* cells after 48‍ ‍h was evaluated as a relative value based on the initial number of cells (Fig. 1A). The number of *Paramecium* cells generally increased in an MOI-dependent manner, with the greatest amount of growth being observed at an MOI of 10^4^. *Paramecium* cells were fed Phi-1 and *K. pneumoniae* at an MOI of 10^4^, and the growth efficiency of *Paramecium* was compared. In our laboratory, *K. pneumoniae* is the standard feed used to culture and maintain *Paramecium* strains. Consequently, *K. pneumoniae*-fed *Paramecium* cells exhibited a nearly 2-fold increase in growth after 48 h, which was significantly higher than that in the non-feeding control group. In contrast, Phi-1-fed *Paramecium* did not exhibit notable growth (Fig. 1B).

### Assessment of the effect on temperature tolerance of *Paramecium* hosts

**Fig. 2.**
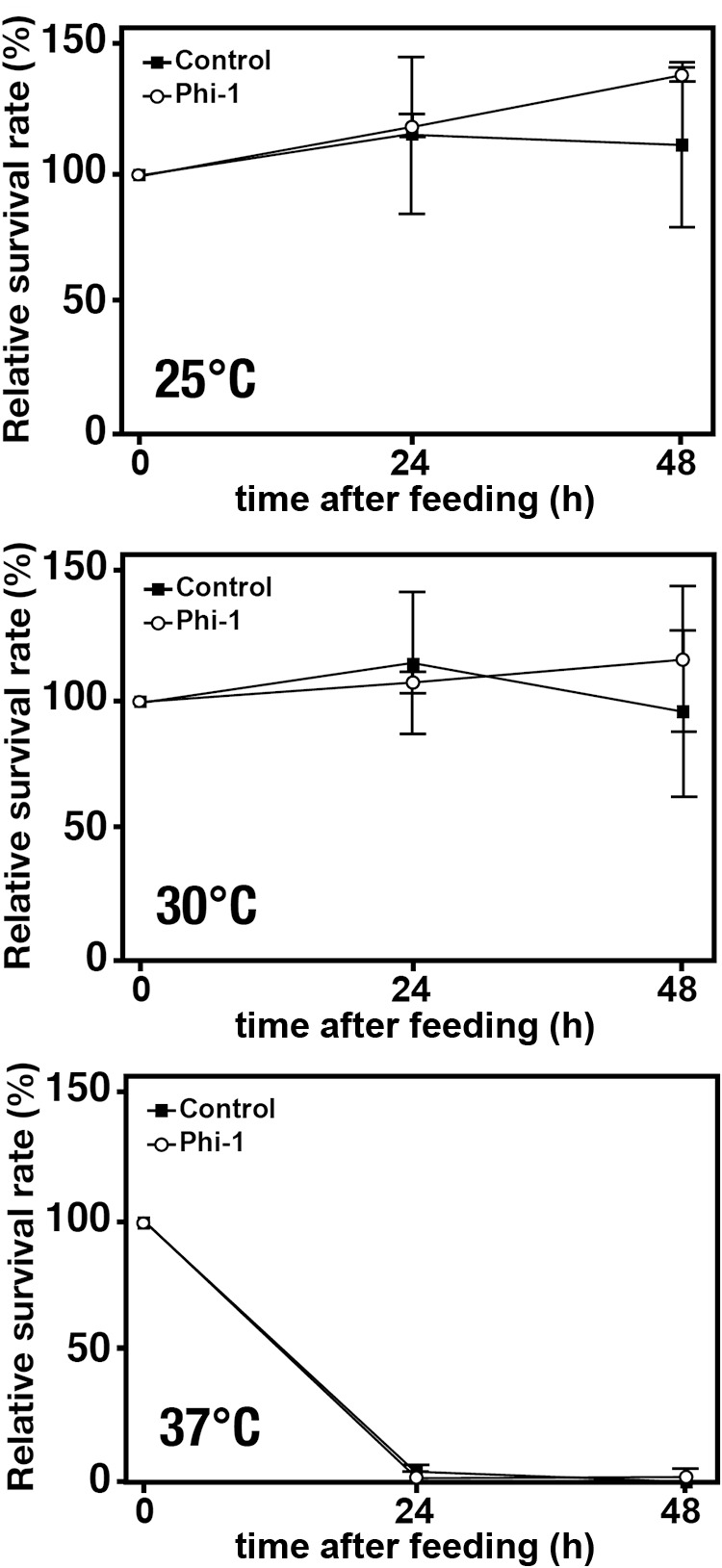
The temperature tolerance of the *Paramecium* host. The relative growth rates of control *Paramecium* cells (without any feeding) and cells fed Phi-1 at an MOI of 10^4^ under different incubation temperatures (25, 30, or 37°C) at 24 and 48‍ ‍h after feeding. Percentages are based on the number of *Paramecium* cells at the beginning of the culture, which was defined as 100%. Data are averages based on samples from three identical experiments, and error bars represent standard deviations.

We investigated whether any benefit was conferred to‍ ‍*Paramecium* cells in the presence of *Legionella*. The optimal growth temperature for *Paramecium* is 25°C. *Paramecium* spp. are tolerant of low temperatures, and may be cultured for extended periods at temperatures ≤10°C. Therefore, a change in resistance to high temperatures following feeding was investigated in *Paramecium*. In comparisons to the control incubation at 25°C, *Paramecium* cells exhibited significant death when incubated at 30°C or 37°C. The same results were obtained when *Paramecium* cells were pre-fed Phi-1 and cultured under the same conditions (Fig. 2). Therefore, symbiosis with *Legionella* did not confer temperature tolerance to *Paramecium* hosts.

### Analysis of cytotoxicity induced by Ofk308 in Phi-1-maintained *Paramecium* cells

**Fig. 3.**
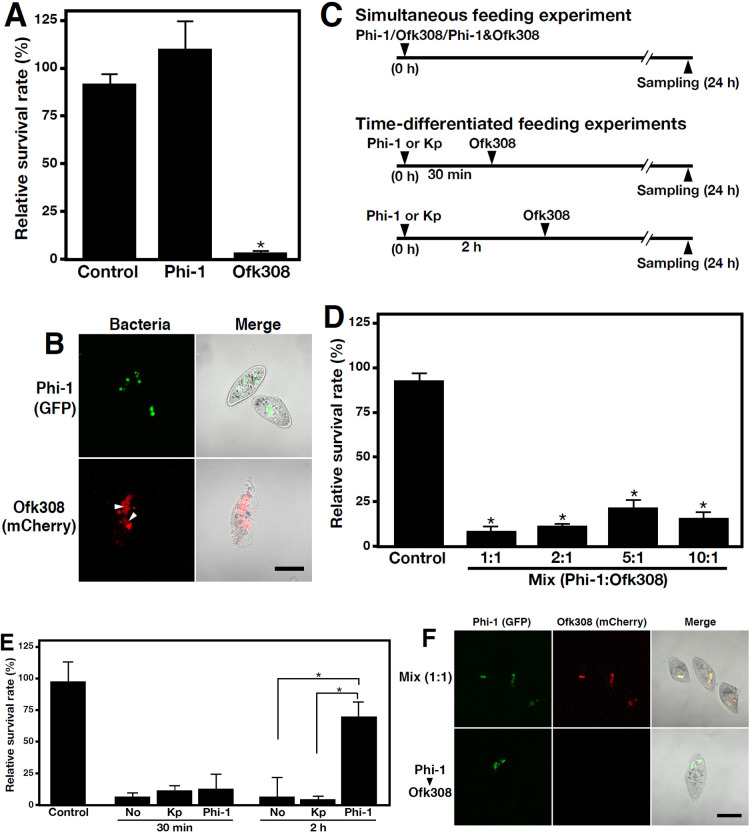
The inhibition of phagocytosis by Phi-1 confers an advantage to *Paramecium* hosts. *Paramecium* cells were fed Phi-1 and Ofk308 (A) or GFP-expressing Phi-1 and mCherry-expressing Ofk308 (B) at an MOI of 10^4^ and were cultured at 25°C for 24 h. Control is the non-feeding group. White arrowheads indicate bacteria that deviate from the phagosome and appear to reside in the cytoplasm of cells with an abnormal morphology. Scale bar, 100‍ ‍μm. (C) Bacteria were added to *Paramecium* cells according to this schedule. (D) *Paramecium* cells were fed both strains at the indicated ratios and were cultured at 25°C for 24 h. Control is the non-feeding group. (E) *Paramecium* cells were fed *Klebsiella pneumoniae* (Kp) or Phi-1 at an MOI of 10^4^, and 30‍ ‍min or 2‍ ‍h later, Ofk308 was fed at the same MOI. The number of surviving cells was assessed after 24‍ ‍h of incubation at 25°C. No; the group fed Ofk308 without any pre-feeding. Control; the group without pre-feeding or Ofk308 feeding. Percentages are based on the number of *Paramecium* cells at the beginning of the culture, which was defined as 100%. Data are averages based on samples from three identical experiments, and error bars represent standard deviations. Significant differences are indicated by asterisks (*P<0.05). (F) *Paramecium* cells were fed a mixture of GFP-expressing Phi-1 and mCherry-expressing Ofk308 at a 1:1 ratio and an MOI of 10^4^ (upper panels) or mCherry-expressing Ofk308 2‍ ‍h after GFP-expressing Phi-1 feeding (bottom panels), and cells were observed after 24 h. Scale bar, 100‍ ‍μm.

Previous studies reported that the presence of a symbiont protected a host from other organisms (Maita *et al.*, 2018; Hrdina *et al.*, 2024). We also demonstrated that the *L. pneumophila* Ofk308 strain, which was isolated from the environment, was cytotoxic in an MOI-dependent manner to specific *Paramecium* strains, including RB-1, which was used in the present study (Watanabe *et al.*, 2016). Therefore, we investigated whether cytotoxicity induced by Ofk308 was modulated in *Paramecium* hosts in the presence of the Phi-1 strain, a non-cytotoxic strain of *L. pneumophila*. We initially confirmed that Ofk308 feeding reduced the number of viable *Paramecium* cells and caused abnormalities in their morphology (Fig. 3A and B). We then conducted an experiment in which *Paramecium* cells were fed Phi-1 and Ofk308 simultaneously at the same MOI (Fig. 3C). The results obtained showed no significant changes in Ofk308 cytotoxicity. Additionally, increasing the ratio of Phi-1 to Ofk308 did not significantly reduce cytotoxicity (Fig. 3D). We then employed an experimental feeding system in which feeding with Phi-1 was performed prior to Ofk308 feeding (Fig. 3C). In the case of Ofk308 feeding 30‍ ‍min after Phi-1 feeding, the reduction in *Paramecium* host cell counts after 24‍ ‍h was similar to that in the group that was not fed Phi-1. However, in the case of Ofk308 feeding 2‍ ‍h after Phi-1 feeding, the reduction in cell counts attributable to Ofk308 cytotoxicity was significantly inhibited (Fig. 3E). This result was not observed in the case of prior feeding with *K. pneumoniae*, suggesting that this effect was specific to *Legionella* feeding. To reveal the mechanisms underlying this result, *Paramecium* host cells were observed in a pattern of simultaneous feeding and a pattern of time-differentiated feeding experiments using Phi-1 and Ofk308, which express different fluorescent proteins. In the case of simultaneous feeding with Phi-1 and Ofk308, Phi-1 and Ofk308 were internalized into *Paramecium* cells to the same degree, and morphological abnormalities were observed. In contrast, when cells were first fed Phi-1, there was virtually no uptake of Ofk308 when it was introduced at later time points and no abnormalities in *Paramecium* cell morphology (Fig. 3F).

### Confirmation of the inhibition of phagocytosis by Phi-1 in *Paramecium*

**Fig. 4.**
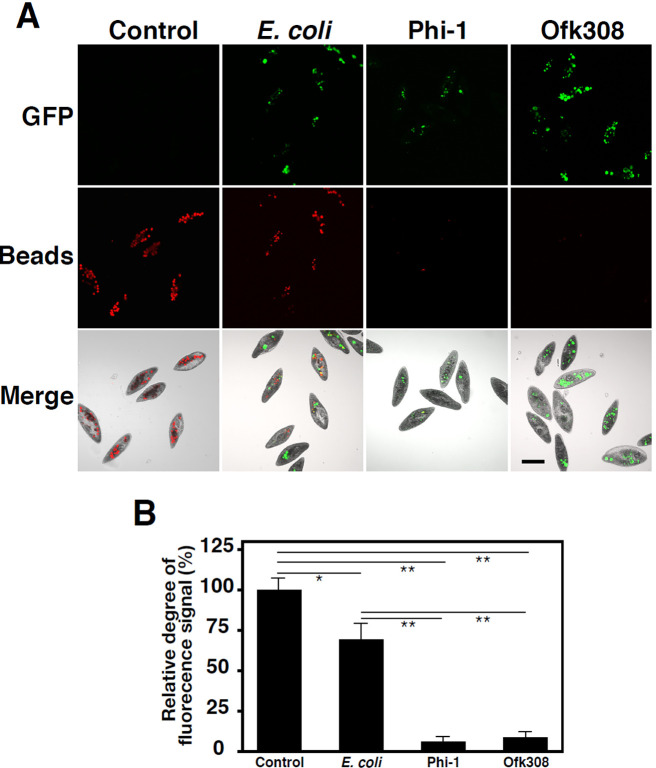
Confirmation of the inhibition of phagocytosis using fluorescent beads. *Paramecium* cells were fed GFP-expressing *E. coli* (Ec), Phi-1, and Ofk308 at an MOI of 10^4^. After 2 h, these cells were incubated with fluorescent beads, followed by the observation of cells 2‍ ‍h later. The microscopic appearance of each *Paramecium* (A) and relative degrees of the fluorescence signal are presented with the results for control *Paramecium* cells (without bacterial feeding) set to 100% (B). Scale bars, 100‍ ‍μm. Error bars represent standard deviations. Significant differences from the control are indicated by asterisks (**P<0.01, *P<0.05).

The results shown in Fig. 3 suggest that phagocytosis and new phagosome formation stopped in *Paramecium* cells that internalized Phi-1. We previously reported that the inhibition of phagocytosis occurred when Ofk308 exhibited cytotoxicity in a different *Paramecium* strain (Nishida *et al.*, 2018). To investigate this in more detail, we performed an experiment to evaluate the phagocytic ability of *Paramecium* using fluorescent beads. GFP-expressing *Legionella* strains of both Phi-1 and Ofk308, as well as *E. coli* as a control, were incubated with *Paramecium* cells, and fluorescent beads were added 2‍ ‍h later. In the control group, which was not fed any bacteria, the normal uptake of fluorescent beads and their accumulation within phagosomes were observed. Similarly, the uptake of beads was noted in *Paramecium* cells fed *E. coli*, but decreased numerically because the intracytoplasmic space was occupied by phagosomes containing *E. coli*. In contrast, in *Paramecium* cells fed both *Legionella* strains, bead uptake was significantly inhibited (Fig. 4A and B).

### Viability ana­lysis of *Paramecium* following phagocytosis inhibition

**Fig. 5.**
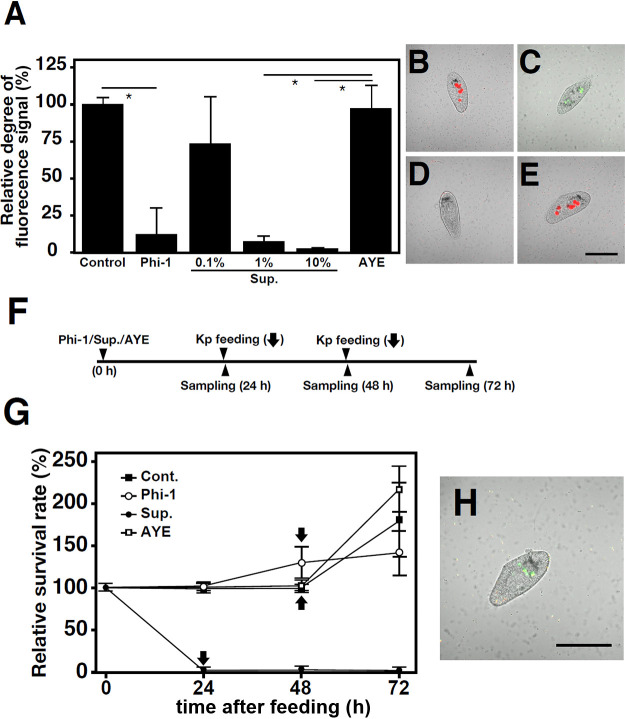
Survival of *Paramecium* cells upon the inhibition of phagocytosis. *Paramecium* cells were fed Phi-1 at an MOI of 10^4^; treated with the culture supernatant of Phi-1 (Sup.) at a final concentration of 0.1, 1, or 10%; or treated with AYE at a final concentration of 10%. After 2 h, these cells were incubated with fluorescent beads, followed by the observation of cells 2‍ ‍h later. Relative degrees of fluorescence are presented with the fluorescence of control *Paramecium* cells (without bacterial feeding) set to 100% (A). Error bars represent standard deviations. Significant differences from the control are indicated by asterisks (*P<0.05). The microscopic appearances of control (B), GFP-expressing Phi-1-fed (C), 10% culture supernatant-treated (D), and 10% AYE-treated (E) *Paramecium* cells are presented. Scale bars, 100‍ ‍mm. (F) Bacteria and culture supernatants were added to *Paramecium* cells according to this schedule. (G) The relative survival rates of control (Cont.), Phi-1-fed (at an MOI of 10^4^), culture supernatant (Sup.)-treated (10%), and AYE-treated (10%) *Paramecium* cells were monitored at the indicated time points. Percentages are based on the number of *Paramecium* cells at the beginning of the culture, which was defined as 100%. Black arrows indicate the timing of additional *Klebsiella pneumoniae* feeding. Data are averages based on samples from three identical experiments, and error bars represent standard deviations. (H) Fluorescent beads were administered 48‍ ‍h after *Paramecium* cells were fed GFP-expressing Phi-1, and imaging was performed 2‍ ‍h later. Scale bars, 100‍ ‍μm.

In our investigation of the mechanism by which *Legionella* inhibit *Paramecium* phagocytosis, the same inhibition was observed when the culture supernatant of *Legionella* was added to *Paramecium* cells (Fig. 5A, B, C, D, and E). Therefore, we exami­ned viability in each group, and found that the majority of *Paramecium* cells treated with the *Legionella* culture supernatant died within 24‍ ‍h (Fig. 5G). Phi-1-fed *Paramecium* hosts remained viable after 48 h, in accordance with the results shown in Fig. 1. Phi-1 was also maintained in the host cells at this time (Fig. 5H). The numbers of *Paramecium* cells in the non-treated control and AYE-treated groups were maintained at 48 h; however, their counts remained lower than those in the Phi-1-fed group. At this point, additional exposure to *K. pneumoniae* as feed resulted in a rapid increase in the number of *Paramecium* cells in the control and AYE-treated groups over the next 24 h, but not in the Phi-1-fed group (Fig. 5G). Relative cell proliferation rates based on the time of additional *K. pneumoniae* feeding were 178.79±41.93, 208.19±20.26, and 109.76±19.26% in the control, AYE-treated, and Phi-1-fed groups, respectively. The Phi-1-fed group exhibited significantly lower values than the other groups. Concurrently, red fluorescent beads were fed to *Paramecium* cells that had been fed Phi-1. *Paramecium* cells were observed 2‍ ‍h later, and few beads were found in the phagosomes of the host cells, thereby confirming that the suppression of phagocytosis was persistent (Fig. 5H).

## Discussion

*Paramecium* act as a natural host for *Legionella* in the environment (Watanabe *et al.*, 2016, 2018). However, the underlying mechanisms and significance of this function remain unclear. The present study focused on the benefits of‍ ‍an established relationship between *Legionella* and *Paramecium*. Previous studies demonstrated that *Legionella* strains are generally not digested by *Paramecium* in the same manner as ordinary bacteria, instead remaining within their phagosomes (Watanabe *et al.*, 2016). As expected from these findings, the present study clearly demonstrated that the value of *Legionella* as a source of nutrition or feed for *Paramecium* was lower than that of other bacteria, such as *K. pneumoniae*, which may be an ideal feed (Fig. 1). We also confirmed that the internalization of *Legionella* did not increase the temperature tolerance of *Paramecium* hosts (Fig. 2). We assumed that if *Paramecium* hosts maintaining *Legionella* acquired some tolerance that leads to an expansion of their living environment, it may also be advantageous for the growth and spread of *Legionella*; however, this was not observed in the present study. Previous studies suggested changes in the tolerance of *Paramecium* cells during the symbiotic relationship with the symbiont *Holospora* spp. (Smurov and Fokin, 1998; Hori and Fujishima, 2003; Fujishima *et al.*, 2005). In comparative genomic ana­lyses between *Legionella* spp. and *Holospora* spp., we identified several homologous genes potentially involved in the establishment of a symbiotic relationship with *Paramecium* hosts (Watanabe *et al.*, 2018). However, even among candidate genes, none appeared to be associated with changes in host tolerance to temperature. Although both bacteria utilize the same *Paramecium* host, *Legionella* remain within phagosomes, and *Holospora* are obligate endonuclear symbionts. Their symbiotic sites and ecology are completely distinct, which may explain their contrasting effects on their hosts.

The present results also showed that the inhibition of phagocytosis was a notable change in *Paramecium* cells after they internalized *Legionella*. We previously reported that Ofk308 inhibited phagocytosis in a TolC-dependent manner in other *Paramecium* strains, and also that this was responsible for cytotoxicity (Nishida *et al.*, 2018). In the present study, this inhibition of phagocytosis was newly demonstrated during Phi-1 feeding (Fig. 4), and the results obtained also confirmed the partial involvement of TolC as well as Ofk308 (Fig. S1). The inhibition of *Paramecium* phagocytosis by *Legionella* has been widely observed across their strains and species, suggesting that this mechanism is involved not only in cytotoxicity to the host, but also in the stable intracellular relationship. Furthermore, the inhibition of phagocytosis, which was insufficient after 30‍ ‍min in an incubation with *Legionella*, was enhanced at 2‍ ‍h (Fig. 3E), suggesting that the effect of the inhibition gradually progressed and that a certain amount of time was required for complete inhibition.

The same results were observed when *Paramecium* cells were treated with the culture supernatant of Phi-1 (Fig. 5A), suggesting that secreted or extracellular components of *Legionella* affected the results obtained. As expected, the treatment with the culture supernatant of Ofk308 also inhibited phagocytosis. Additionally, similar effects were observed for the culture supernatants of *dotA*, a T4SS-deficient mutant, and *tolC* mutant strains (Fig. S1). These results indicate that the inhibition of phagocytosis by culture supernatants of *Legionella* may be a common event in various strains. Furthermore, this inhibition may be induced by a novel factor that is independent of T4SS. Moreover, TolC-dependent inhibition may be observed when *Legionella* exist within *Paramecium* cells and do not play a role in the inhibitory effect of the culture supernatant. To identify the factors in the culture supernatant that are responsible for this effect, Phi-1 culture supernatants were fractionated by‍ ‍mo­lecular weight, treated with protease, or treated with heat; however, none of these treatments reduced the inhibitory effect on phagocytosis (Fig. S2). Although it is currently not possible to exclude the hypothesis that this effect is due to a deviant component from dead bacteria, the involvement of a non-protein component or some type of low-mole­cular-weight component is strongly suggested. If these factors were actively and strategically synthesized or secreted by *Legionella*, it may be significant for *Legionella* as symbionts to be able to prevent the visitation of new competitors and to use host cells exclusively. Since this competition between different symbionts has been reported in other organisms (Guckes and Miyashiro, 2023; Wang *et al.*, 2023), it may also occur in relationships in *Paramecium* hosts.

The suppression of phagocytosis by maintaining *Legionella* may be advantageous for the host in terms of preventing the invasion or uptake of harmful organisms. The present study experimentally demonstrated the host-side advantage in feeding assays utilizing Ofk308, which are cytotoxic to *Paramecium* hosts (Fig. 3). The maintenance of Phi-1 promoted the viability of *Paramecium* hosts by inhibiting the cellular uptake of Ofk308. These protective effects have been reported in other host species. For example, *Neochlamydia* protect their host amoebae against *Legionella* infection (Maita *et al.*, 2018). Several factors have been implicated in the cytotoxicity of Ofk308 against *Paramecium* hosts (Watanabe *et al.*, 2016; Nishida *et al.*, 2018). The present study also demonstrated that Ofk308 did not exhibit cytotoxicity unless it reached the intracellular space or phagosomes of *Paramecium* cells (Fig. 3B and F). These results support our previous hypotheses regarding the mechanisms underlying and factors contributing to Ofk308-induced cytotoxicity.

However, it is important to note that the prevention of non-specific phagocytosis also means the suspension of new feed uptake. This may be a significant disadvantage to *Paramecium* hosts. In other words, *Paramecium* hosts may starve to death by maintaining *Legionella*. In an experiment in which the similar inhibition of phagocytosis was induced by the administration of a *Legionella* culture supernatant, the majority of *Paramecium* cells died within 24‍ ‍h (Fig. 5). The death of *Paramecium* cells may be attributed to factors other than the induction of starvation. However, no changes were observed in *Paramecium* motility or morphology 2‍ ‍h after the addition of the supernatant, indicating that the supernatant contained a potent and fast-acting cytotoxic component and also that *Paramecium* were not immediately killed within minutes to hours due to this effect (Fig. S3). In contrast, the intracellular presence of live *Legionella* Phi-1 in *Paramecium* hosts did not result in starvation despite the prolonged inhibition of phagocytosis. Instead, a small amount of growth was observed (Fig. 1 and 5). These results suggest that intracellular *Legionella* are at least involved in maintaining life to the extent that they keep *Paramecium* hosts alive for several days without starvation. It is not possible to exclude the possibility that a very small number of *Paramecium* did not have internalized *Legionella* and, thus, continued to have a normal phagocytic function. Additionally, it is conceivable that minor *Paramecium* may have internalized supplementary *K. pneumoniae* fed 48‍ ‍h later as a source of nutrients, thereby exhibiting active cell division; however, the impact on the overall number of *Paramecium* cells would be insignificant. Previous studies reported the involvement of symbionts in the regulation of the host’s metabolic system or in directly providing nutritional benefits through their own metabolites (Douglas, 2018; Newton and Rice, 2020; da Silva Soares *et al.*, 2023). In the case of *Paramecium*, *P. bursaria* provides carbon dioxide and nitrogen to its symbiont *Chlorella*. In return, *Chlorella* performs photosynthesis and provides oxygen and sugars obtained through photosynthesis to host *P. bursaria* cells, resulting in a mutually beneficial “symbiotic” relationship (Brown and Nielsen, 1974; Reisser, 1976a, 1976b). A similar relationship has not been reported for *Paramecium* hosts and bacterial symbionts or been identified between *Legionella* and protist hosts other than *Paramecium*. Future studies to elucidate the mole­cular mechanisms by which *Legionella* provide nutritional benefits to *Paramecium* hosts may provide indisputable evidence to show that *Legionella* are beneficial symbionts for *Paramecium* and, thus, their relationship is‍ ‍mutualistic symbiosis. Furthermore, the present results were based on experiments utilizing a restricted set of *Paramecium* and *Legionella* strains. To reach a broader conclusion regarding the relationship between *Paramecium* and *Legionella*, it is necessary to examine a larger number of species and strains in future studies.

## Citation

Kiyota, H., Watanabe, K., Oyama, H., Tachibana, M., Shimizu, T., and Watarai, M. (2025) *Legionella* Confer Survival Benefits to *Paramecium* Hosts by Inhibiting Phagocytosis. *Microbes Environ ***40**: ME25022.

https://doi.org/10.1264/jsme2.ME25022

## Supplementary Material

Supplementary Material
