# Supplementary material for: Legionella Confer Survival Benefits to Paramecium Hosts by Inhibiting Phagocytosis
Source: Microbes Environ. 2025 Oct 31;40(4):ME25022. doi: 10.1264/jsme2.ME25022 (PMC12727205; doi:10.1264/jsme2.ME25022)
Supplement: Supplementary file 1 — Supplementary Material [file 40_25022_s1.pdf]

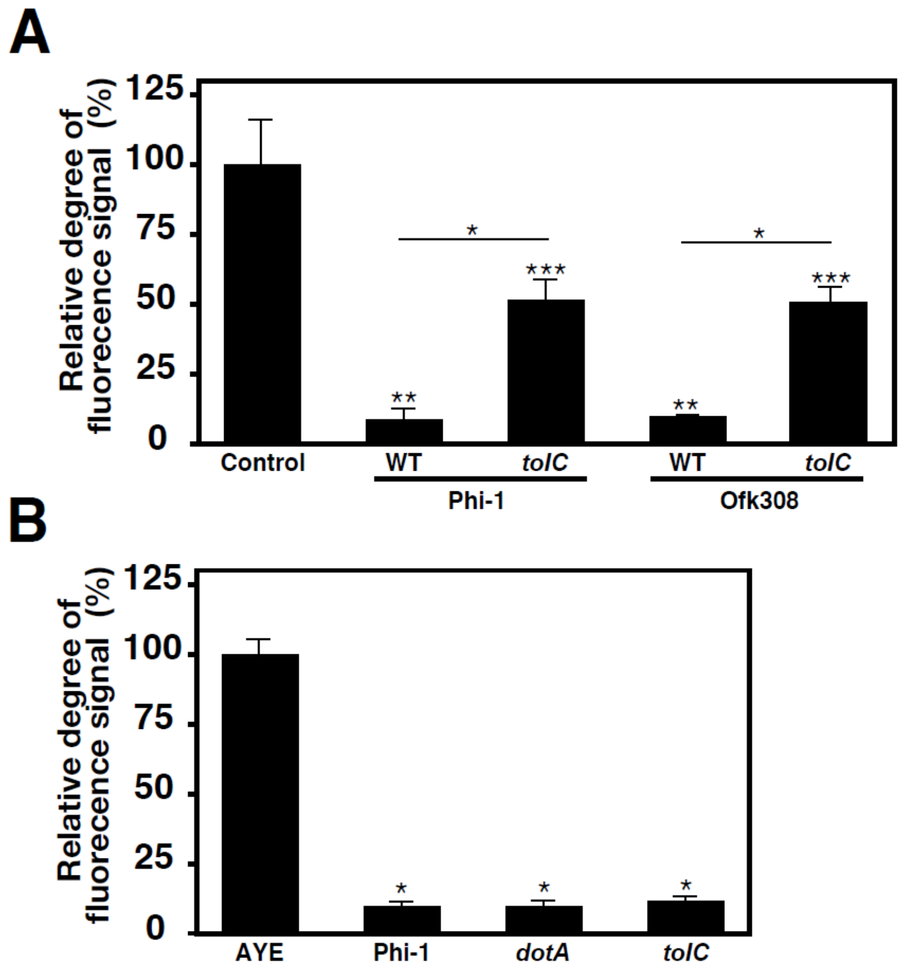

Supplemental Figure S1.

(A) *Paramecium* cells were first fed with Phi-1, *tolC* mutant of Phi-1, Ofk308, and *tolC* mutant of Ofk308 at an MOI of 10^4. Two hours later, these cells were incubated with fluorescent beads, followed by observation of the cells 2 h later. Relative degrees of fluorescence signal are presented with the findings for control Paramecium cells (without bacterial feeding) set to 100%. Error bars represent standard deviations. Statistically significant differences between Wild type (WT) and mutant strains (* $P < 0.01$) and compared with the control (** $P < 0.01$, *** $P < 0.05$) are indicated by asterisks. (B) *Paramecium* cells were first treated with the culture supernatant of Phi-1, *dotA* mutant, and *tolC* mutant at a final concentration of 10%. Two hours later, these cells were incubated with fluorescent beads, followed by observation of the cells 2 h later. Relative degrees of fluorescence are presented with the fluorescence of *Paramecium* cells treated with AYE set to 100%. Error bars represent standard deviations. Statistically significant differences compared with AYE are indicated by asterisks (*$P < 0.01$).

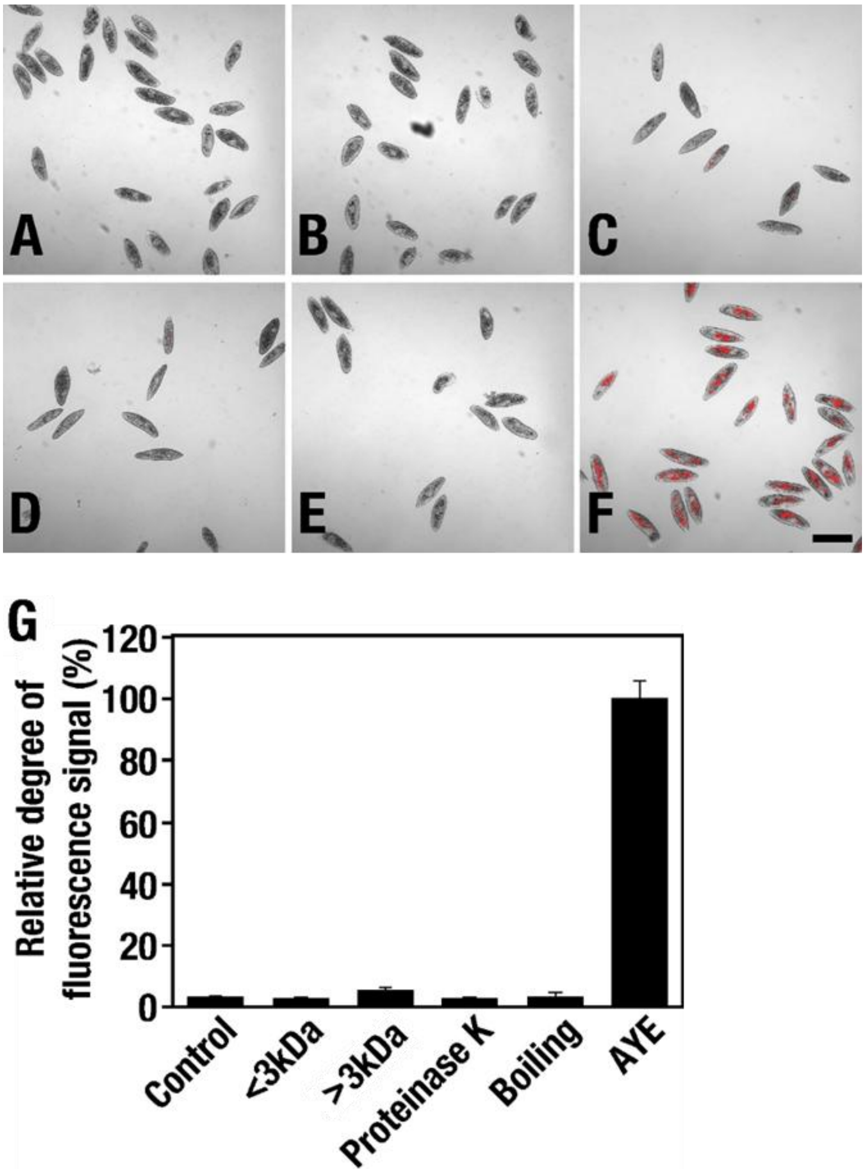

Supplemental Figure S2.

Paramecium cells were first administered with Phi-1 culture supernatant without treatment (Control, A), of < 3 kDa fraction (B), of 3 > kDa fraction (C), treated with proteinase K (D), treated with heat (E), and AYE medium (F) at a final concentration of 10%. Two hours later, these cells were incubated with fluorescent beads, followed by observation of the cells 2 h later. Relative degrees of fluorescence signal are presented with the findings for AYE-administered *Paramecium* cells set to 100% (G). Scale bars, 100 µm. Error bars represent standard deviations.

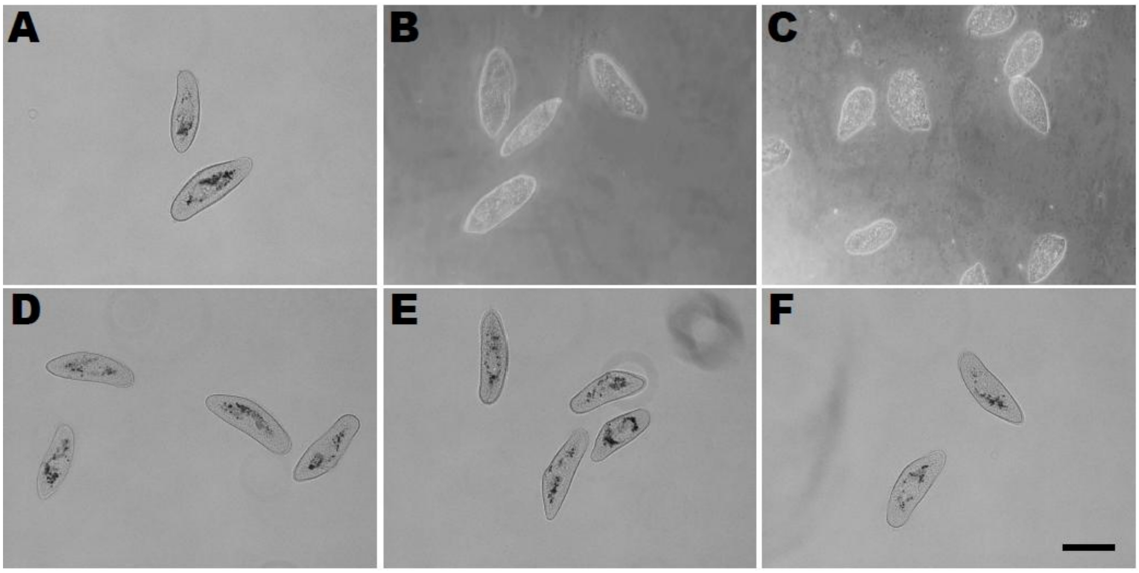

Supplemental Figure S3.

Paramecium morphology was observed 2, 12, and 24 h after the administration of culture supernatant (A:2 h, B:12 h, and C: 24 h) or AYE (D: 2 h, E:12 h, and F: 24 h) at a final concentration of 10%. Scale bars, 100 µm.
